# Toward Patient-Specific Prediction of Ablation Strategies for Atrial Fibrillation Using Deep Learning

**DOI:** 10.3389/fphys.2021.674106

**Published:** 2021-05-26

**Authors:** Marica Muffoletto, Ahmed Qureshi, Aya Zeidan, Laila Muizniece, Xiao Fu, Jichao Zhao, Aditi Roy, Paul A. Bates, Oleg Aslanidi

**Affiliations:** ^1^School of Biomedical Engineering and Imaging Sciences, King's College London, London, United Kingdom; ^2^Biomolecular Modelling Laboratory, The Francis Crick Institute, London, United Kingdom; ^3^Auckland Bioengineering Institute, University of Auckland, Auckland, New Zealand; ^4^Department of Computer Science, University of Oxford, Oxford, United Kingdom

**Keywords:** atrial fibrillation, patient imaging, catheter ablation, computational modelling, deep learning, classification algorithm

## Abstract

Atrial fibrillation (AF) is a common cardiac arrhythmia that affects 1% of the population worldwide and is associated with high levels of morbidity and mortality. Catheter ablation (CA) has become one of the first line treatments for AF, but its success rates are suboptimal, especially in the case of persistent AF. Computational approaches have shown promise in predicting the CA strategy using simulations of atrial models, as well as applying deep learning to atrial images. We propose a novel approach that combines image-based computational modelling of the atria with deep learning classifiers trained on patient-specific atrial models, which can be used to assist in CA therapy selection. Therefore, we trained a deep convolutional neural network (CNN) using a combination of (i) 122 atrial tissue images obtained by unfolding patient LGE-MRI datasets, (ii) 157 additional synthetic images derived from the patient data to enhance the training dataset, and (iii) the outcomes of 558 CA simulations to terminate several AF scenarios in the corresponding image-based atrial models. Four CNN classifiers were trained on this patient-specific dataset balanced using several techniques to predict three common CA strategies from the patient atrial images: pulmonary vein isolation (PVI), rotor-based ablation (Rotor) and fibrosis-based ablation (Fibro). The training accuracy for these classifiers ranged from 96.22 to 97.69%, while the validation accuracy was from 78.68 to 86.50%. After training, the classifiers were applied to predict CA strategies for an unseen holdout test set of atrial images, and the results were compared to outcomes of the respective image-based simulations. The highest success rate was observed in the correct prediction of the Rotor and Fibro strategies (100%), whereas the PVI class was predicted in 33.33% of the cases. In conclusion, this study provides a proof-of-concept that deep neural networks can learn from patient-specific MRI datasets and image-derived models of AF, providing a novel technology to assist in tailoring CA therapy to a patient.

## 1. Introduction

Atrial fibrillation (AF) is the most common cardiac arrhythmia and is characterised by rapid and uncoordinated contraction of the atria. It is associated with high levels of morbidity and is the leading cause of stroke in people over 75 (Hart and Halperin, 2001). Although the precise mechanisms underlying AF remain unclear, it has been recognised that ectopic electrical beats originating from the pulmonary veins (PVs) can trigger AF (Chen et al., 1999), and that electrical rotors generated by breakdown of such ectopic waves provide self-sustained drivers for AF. In addition, areas of fibrotic atrial tissue have been linked with slow conduction of electrical waves, providing anchoring points for the rotors, and thus arrhythmogenic locations in the atria (Morgan et al., 2016; Roy et al., 2020).

First line clinical treatments for AF include antiarrhythmic drugs, electrical cardioversion, and catheter ablation therapy (Parameswaran et al., 2021). Radiofrequency catheter ablation (RFCA) involves controlled destruction of arrhythmogenic locations via delivery of localised RF energy to atrial tissue through a catheter. RFCA procedures have a relatively high success rate in patients with paroxysmal AF (about 70% for a single procedure) (Oketani et al., 2012). However, in persistent AF patients, the arrhythmia can recur after RFCA in ~75% of cases (Wang et al., 2017). Cryo-ablation has emerged as an alternative, arguably more convenient method based on delivery of low temperatures through a balloon catheter. However, clinical trials have shown no significant difference in long-term efficacy of RF ablation vs. cryo-ablation of paroxysmal AF patients (Andrade et al., 2019). This warrants the development of novel, more efficient ablation strategies (Gong et al., 2015).

RFCA creates lines of conduction block on the atrial surface, which should ideally have minimal length and allow for quick recovery of the mechanical activity of both atria during sinus rhythm (Ruchat et al., 2007). The only clinically proven empirical strategy is Pulmonary Vein Isolation (PVI), which generates circumferential lesions around the right and left PVs. Promising novel strategies include rotor- and fibrosis- driven CA (Parameswaran et al., 2021). The former targets focal points of electrical activation to terminate rotors (Zaman et al., 2015), while the latter aims to minimise the effect of fibrosis by applying box isolation of fibrotic areas (BIFA) (Schreiber et al., 2017) or linear lesions across fibrotic tissue (Kottkamp et al., 2017).

The heterogeneous results obtained by different studies suggest that a single ablation strategy is unlikely to be successful for all patients, and the improvement of CA therapy can come from personalised approaches to each patient. The latter have successfully applied patient medical imaging and image-based computational modelling (Boyle et al., 2019). We aim to simulate various scenarios of AF and personalised CA strategies using computational models on atrial tissue based on patient imaging data, and use the model simulation data to train deep convolutional neural networks (CNNs), which have proven to be successful for biomedical problems (Pfeiffenberger and Bates, 2018; Poh et al., 2018). Once the CNN is trained, we will use it to identify patient-specific patterns of CA lesions for each scenario.

We have previously developed a method for training CNNs on a set of synthetic data composed of 2D atrial tissues with randomly distributed fibrotic patches (Muffoletto et al., 2019). To prove its effectiveness and provide proof-of-concept results for patients, we will apply this approach to patient-specific 2D atria obtained by unfolding of 3D atrial datasets from AF patients. The latter have been obtained using late gadolinium enhancement-magnetic resonance imaging (LGE-MRI) (Williams et al., 2017; Xiong et al., 2021), which is primarily used to image cardiac fibrosis. LGE-MRI scans are routinely performed before CA procedures in many clinical centres, and hence, LGE data represents a perfect reference for studies of patient-specific AF scenarios and ablation patterns.

## 2. Materials and Methods

### 2.1 Patient-Specific Atrial Tissues

The patient-specific dataset was obtained from two sources. The first dataset, from the Atrial Segmentation Challenge at the Statistical Atlases and Computational Modelling of the Heart 2018 workshop (Xiong et al., 2021), consisted of 86 LGE-MRI scans from patients with AF (original resolution of 0.625 fnins-15-654170-i0001 0.625 fnins-15-654170-i0001 0.625 mm^3^), and included the corresponding 3D left atria (LA) segmentations. The second dataset was collected at St Thomas' Hospital (Chubb et al., 2018) from 18 AF patients pre- and post-CA, providing total 36 LGE-MR images (original resolution of 1.3 fnins-15-654170-i0001 1.3 fnins-15-654170-i0001 4 mm^3^, reconstructed to 0.94 fnins-15-654170-i0001 0.94 fnins-15-654170-i0001 2 mm^3^).

**Figure 1:**
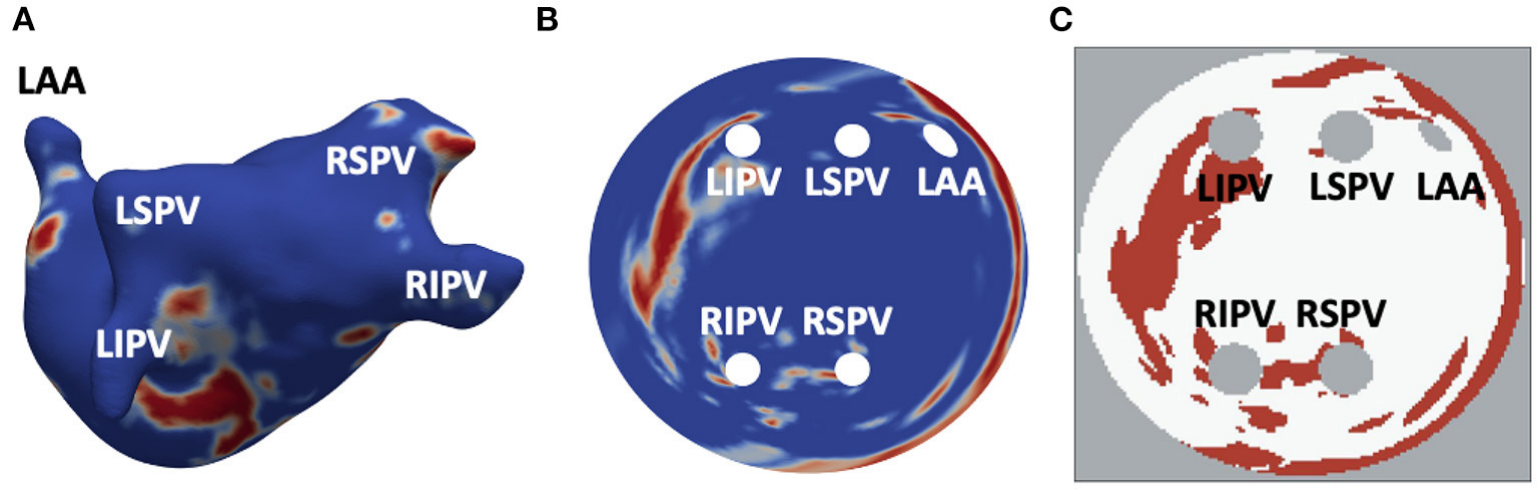
Generation of 2D LA tissue images from LGE-MRI data. **(A)** 3D LA geometry segmented from LGE-MRI patient data, with LGE-MR image intensity showing healthy myocardium (blue) and regions of fibrosis (red). **(B)** 2D LA-SUM representation where 3D LA has been unwrapped to a standardised disk form. The PVs and LAA are situated in the centre while the mitral valve becomes the border of the disk. **(C)** Thresholded LA-SUM images. White represents healthy tissue, red denotes fibrotic regions, while grey indicates areas of empty space (background and openings of the PVs and LAA). PVs and LAA are labelled in all figures.

All LGE-MR images were segmented based on the image intensity distributions, to enhance visualisation of atrial fibrosis for the improved inter-patient quantification and comparisons. To do this, an image intensity ratio (IIR) value was calculated for each voxel on the 3D model by division of LGE intensity by mean blood pool intensity. If the IIR value exceeded an empirical threshold of 1.24, the voxel was labelled as dense fibrotic tissue (red in Figures 1A–C) while an IIR of below 1.08 indicated healthy tissue (blue in Figures 1A,B) (Roy et al., 2018). Applying the standardised segmentation method to all LGE-MRI datasets created a set of patient-specific 3D LA models with fibrosis.

The LA models were unfolded to a standardised 2D disk representation for further processing and simulation. This was carried out using the LA standardised unfold mapping (LA-SUM) technique (Williams et al., 2017). Each model was registered to a reference atlas mesh and unfolded by mapping of features from the 3D mesh to their corresponding locations on the 2D LA disk (standard diameter 150, resolution 0.3 fnins-15-654170-i0001 0.3 mm^2^) (Qureshi et al., 2020), with the four PVs and LAA inside and the mitral valve becoming the border of the disk. After the unwrapping, LGE MRI intensities in the 2D LA disks were thresholded to produce binary 2D images with healthy myocardium and fibrotic patches (Figure 1C). Figure 1 illustrates the entire process and the correspondence of unwrapped and thresholded 2D LA disks with the real atria.

### 2.2. Synthetic Atrial Tissues

Since CNNs are contingent on big data and susceptible to overfitting, a set of data augmentation techniques was implemented to expand the limited real dataset. Given that the 3D LA models were unwrapped onto standardised 2D disks, disparities between them were solely due to the fibrosis regions, with no variance of the PVs size and position in the dataset. Hence, an additional set of synthetic 2D LA tissue models with varying fibrosis distributions and PV positions and sizes was generated from the real data.

The generation of synthetic tissues was a three-staged process, which first involved a weighted averaging of 65 real 2D LA images randomly extracted from the STACOM 2018 dataset, which was the larger of the two datasets, and hence could provide greater variability in the fibrosis distributions. This was done by giving a random weight between 0 and 1 (i.e., intensity of each voxel was multiplied by this random weight). With this technique, a dataset of unique 157 synthetic tissues was generated.To ensure even more variability within the fibrosis distributions of these tissues, a second stage followed. For each of the new tissues, the fibrosis distribution was first extracted and thresholded and successively augmented using one or multiple of three affine transformations: flipping, rotation, or translation. The value of threshold and the types of transformation per tissue was selected by randomly assigning to each of them one of 10 cases with different combinations of threshold (ranging from 0.065 to 0.095) and transformations.

Finally, the last stage was performed to introduce variation for the PVs. In half of the synthetic images the size and positions of PVs were altered by randomly assigning one of 6 different variants. For variants 1–3, only the sizes of the PVs were modified by randomly varying their original diameter between 5 and 50%. For variant 1, only one random value was used to resize all four PVs. For variant 2, two random values were chosen, one for the left pair of PVs (LIPV and LSPV), and another for the right pair (RIPV and RSPV). For variant 3, four random values were chosen for each PV. For variants 4–6, in addition to varying the PV size, positions of the PVs were randomly varied in the horizontal and vertical directions.

### 2.3. Atrial Tissue Model

The 2D atrial tissues described in the previous sections (122 real and 157 synthetic) were used to simulate electrical activity sustaining AF and suitable personalised successful strategies for each tissue. Thus, a unique 279 strong dataset was created to combine the 2D LA images and labels assigned to each image after the simulations of AF and ablation. This provided a versatile and robust set to train, test, and validate our neural network classification algorithm (see section 2.3 below).

Propagation of electrical activation waves in LA tissue was simulated using the standard monodomain equation:

(1) $$\begin{array}{r} \frac{\partial V_{m}}{\partial t}=\nabla \cdot \text{D}\nabla V_{m}-\frac{I_{ion}}{C_{m}} \end{array}$$

Here, *V*_*m*_ represents the membrane voltage, *C*_*m*_ is the specific cell capacitance, and **D** is the diffusion tensor that characterises electrical coupling in the tissue. For isotropic tissue, the latter is a constant. Equation (1) was solved using forward Euler method with a time step of 0.01 ms, combined with finite difference approximation of the Laplacian with a spatial step of 0.3 mm.

For simulation of the ion current, *I*_*ion*_ the Fenton-Karma semi-physiological model was applied. The model uses three currents to represent the main ionic currents responsible for the electrical activation and inactivation dynamics of atrial cells. These are the fast inward (flow of *Na*^+^), the slow inward (flow of *Ca*^2+^) and slow outward (flow of *K*^+^) currents:

(2) $$\begin{array}{r} I_{ion}=I_{fi}+I_{so}+I_{si} \end{array}$$

All the currents were implemented using the standard equations and parameters, as previously described (Roy et al., 2018).

Equations (1 and 2) describe the propagation of electrical waves through atrial tissue. To generate the wave breakdown leading to re-entrant drivers (rotors), the standard cross-field protocol was used (Tobón et al., 2014). The rotor was defined as a stable re-entrant circuit lasting for at least 2,000 ms. Trajectories of such rotors can be tracked in the 2D and 3D atrial models even if they hyper-meander (Roy et al., 2020). Only stable rotors were used to simulate AF (see below).

**Figure 2:**
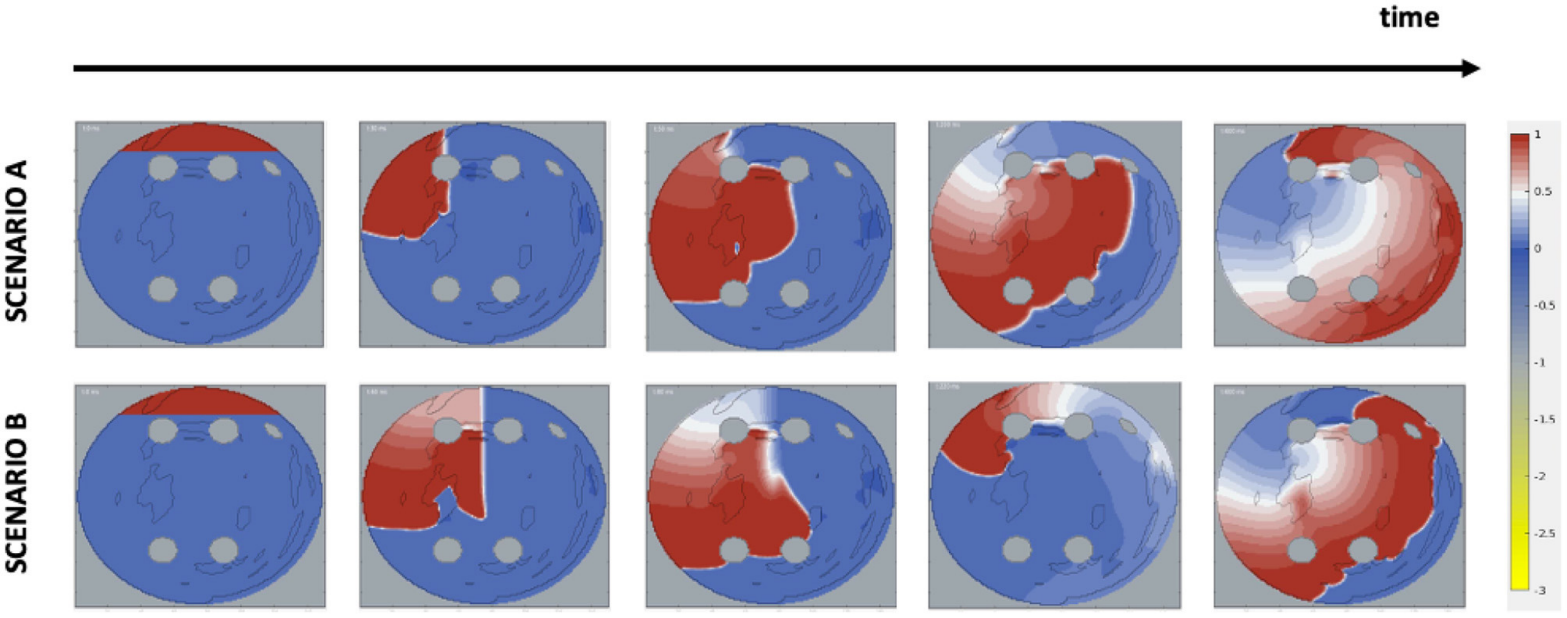
Initiation of AF scenarios in 2D LA tissue models. The images show the voltage distribution (normalised to 0–1 range) in the 2D LA and its changes over time, and the formation of rotors in two scenarios. Each time frame shows, respectively: start of the simulation, application of the cross-field protocol, initial formation of a wave phase break, stabilisation of the rotor and the end of the simulated episode. In Scenario A, the wave is cut off after 28 ms and at the left top pulmonary vein; in Scenario B, the wave is cut off later at 58 ms and at the centre of the tissue.

Two AF scenarios were simulated for each 2D LA tissue model, as illustrated in Figure 2. In Scenario A, the cross-field protocol was applied after 28 ms from the start of the simulation and on the LSPV, and *D* in the healthy myocardium was set to 0.1 mm^2^ms^−1^, producing wave velocity of 0.7 m/s typical of early-stage AF. In Scenario B, the cross-field protocol was applied after 58 ms from the start of the simulation and at the centre of the tissue, with *D* in healthy tissue set to 0.05 mm^2^ms^−1^, producing velocity of 0.5 m/s typical of persistent AF. In some cases where the cross-field protocol didn't result in sustained re-entry, the cross-field application time was increased, so that AF became sustained. The diffusion coefficient in fibrotic areas for both scenarios was set to 0.15 fnins-15-654170-i0001 *D* to simulate slow conduction. For areas corresponding to PVs and LAA, both the diffusion coefficient and membrane potential were set to zero. The simulations of two AF scenarios for each atrial image effectively doubled the size of the dataset, bringing the total number of image-based 2D atrial models in the dataset from 279 to 558 (244 real and 294 synthetic images).

Ablation lesions were simulated by setting values of the membrane potential and diffusion coefficient to zero in small circular areas corresponding to a catheter tip touching the tissue; zero-flux boundary conditions were applied around such areas, as well as the PVs and LAA.

The rotor's tip—a focal point of its rotations—was tracked during the simulations. The tips were identified as the intersection of isolines of *V*_*m*_ and its time derivative (Fenton and Karma, 1998; Roy et al., 2020):

(3) $$\begin{array}{r} V\left(\text{r},n\Delta t\right)=V\left(\text{r},\left(n+1\right)\Delta t\right)=V_{iso} \end{array}$$

The value of *V*_*iso*_ used in the tracking was 0.8.

### 2.4. Tissue Labelling Process

**Figure 3:**
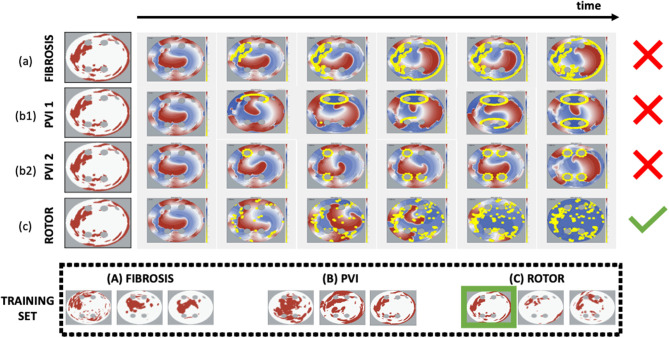
Simulations and identification of successful CA strategy for each 2D LA tissue model. Each CA strategy **(a)** Fibro, **(b1)** PVI 1, **(b2)** PVI 2, **(c)** Rotor was simulated twice (at 10 and 30 ms ablation intervals). The strategy was defined as successful if at least in one of the two trials AF was terminated by the end of the simulation. The horizontal arrow shows the time axis and the six panels under it are the voltage distributions in 2D LA tissue for successive moments of time, where the yellow dots represent the ablated points. The last panel (end of simulation) reveals the difference in ablation patterns between the completed strategies. The green tick indicates CA success, while the red cross indicates failure. In the example shown, the Rotor strategy appears to be the only successful one for the sample tissue, and hence this set goes straight in the Rotor class. The bottom panels (under Training Set) show several examples of labelled tissues derived from this process.

All 2D LA tissue images were labelled to represent common CA strategies: (a) fibrosis-based, (b) PVI, and (c) rotor-based ablation (Parameswaran et al., 2021). Each strategy was simulated in the respective 2D LA tissue models. The simulations for each strategy are illustrated in Figure 3. For fibrosis-based ablation (BIFA), the border between healthy and fibrotic tissue was ablated. For PVI, two approaches were used: one ablating each individual PV, and another applying two large lesions around the left and right pair of PVs (Parameswaran et al., 2021). For rotor-based ablation, the tip of the rotor was identified (Equation 3) and ablated. Each strategy was simulated twice, using 10 and 30 ms intervals between applying successive ablation lesions. The simulation duration was restricted to 2,000 ms and the maximum percentage of ablated tissue was restricted to 40%. Note that most simulations were not performed until 40% of the tissue was ablated, with the rotors terminated by ablation at a much earlier stage; this condition was introduced to avoid ablation by critical mass reduction at the expense of substantially damaging the LA, and practically was only fulfilled in a small number of cases. At the end of the simulation, the CA strategy was defined as successful if at least in one of the two trials AF is terminated. Figure 3 illustrates the assignment of a label for a sample 2D LA tissue based on a unique identification of successful CA strategy.

Two special cases were taken into consideration when assigning the labels: (1) no CA success was reached in simulations of some specific 2D LA models, (2) multiple CA strategies worked in simulations of the same 2D LA model. In case 1, the tissues with no identifiable label were discarded, reducing the initial dataset of 558 samples to 457 (Scenario A+B). To account for case 2, two different labelling methods were utilised. In the first, further referred to as the minimum percentage method, the label was assigned based on the successful CA strategy that resulted in the minimum percentage of ablated tissue. The second, referred to as class availability method, was introduced to make the training set more balanced by prioritising CA strategies that were scarcely represented in the set: first PVI, and then fibrosis-based ablation over the predominant rotor-based. A label generally represented the most successful ablation strategy for a given image/image-based 2D atrial model, but for some images more than one CA strategy was successful in terminating AF. In such cases, the label was chosen with the aim to create the most balanced training set: PVI was the smallest class, and hence the PVI label was assigned preferentially over Fibro and Rotor labels; Fibro was the second smallest class, and hence it was assigned preferentially over Rotor.

The breakdown of successful CA strategies, labelled using each of the two methods described above, is shown in Table 1 for AF Scenarios A and B (see section 2.1 above) and a combination of both (A+B).

**Table 1:** Distribution of labelled 2D LA datasets by scenario (A,B,A+B), labeling method (minimum percentage and class availability), and label class (PVI, Fibro, and Rotor).

**Scenario**	**Labelling method**	**Classes**		
		**PVI**	**FIBRO**	**ROTOR**
Scenario A
	min percentage	40(+4)	38(+4)	136(+4)
	Class availability	64(+6)	70(+5)	80(+1)
Scenario B
	Min percentage	35(+5)	38(+3)	143(+7)
	Class availability	74(+6)	58(+3)	84(+6)
Scenario A+B
	Min percentage	75(+9)	76(+7)	279(+11)
	Class availability	138(+12)	128(+8)	164(+7)

*The main values correspond to the training and validation dataset, while smaller values in brackets correspond to the respective numbers of a separate test set*.

### 2.5. Convolutional Neural Network

To identify CA patterns, a classification algorithm was applied to the labelled 2D LA tissues. The algorithm was constructed using TensorFlow (Abadi et al., 2016), in conjunction with the Python Keras library (Gulli and Pal, 2017; Chauhan and Ram, 2018).

The CNN architecture consisted of four 2D convolutions made of 32 filters of size 3 fnins-15-654170-i0001 3, followed by Rectified Linear Unit (ReLU) activation and using a maxnorm constraint. The convolution block was followed by a MaxPooling of pool size 2 fnins-15-654170-i0001 2, a Flatten layer, and two Dense layers, the first of which was made of 512 units and had ReLU activation and maxnorm kernel constraint. This was followed by a Dropout layer at rate 0.9 and a softmax function. Due to the imbalance of classes we employed the class weighting technique, available in the Keras open-source library as an extra model parameter. This weights the loss function during training based on the percentage of samples in each class. The class weights were found for each model using a scikit-learn package function based on technique inspired by King and Zeng (2001). As suggested in Valova et al. (2020), we used the Adam optimiser and a decay of 0.8 when the validation loss, monitored at every 200 epochs, did not show significant (1*e*^−3^) decrease.The maximum number of epochs was set to 500, but the best classifier was usually found at an earlier stage.

Some of the architecture characteristics (number of conv2D layers, filter size) and hyperparameters (dropout rate, optimiser initial learning rate, optimiser decay, rate of change patience) were extensively tested before setting the final values listed above and these results are shown in the (Supplementary Figure 1).

Classifiers were trained on 2D LA tissue models with simulated (1) Scenario A only, (2) Scenario B only, and (3) a combination of the two scenarios A and B, that we refer to as Scenario A+B. Scenarios A and B were simulated separately, and labels were assigned to images based on each scenario separately. The CNN was then also trained separately, once for scenario A, and once for scenario B. The combined scenario A+B did not involve re-running the simulations or re-labelling the images, the CNN was trained using labels produced in both these scenarios. However, since using the same image with two different labels would result in poor training of the CNN, an original binary image was used with a label from scenario A, and an inverted binary image (“negative”) was used with a label from scenario B. It's important to note that 2D LA images used as the CNN inputs are the same in both Scenario A and B, and only difference in the inputs comes from the ground truth labels—successful CA strategies are different between the scenarios due to the change in AF initiation protocol.

A stratified five-fold cross validation was applied with a train-validation split of 80–20%. Prior to the splitting, 27 sets (9 real + 18 synthetic) were excluded from the original dataset to carry out separated testing on unseen samples. This was performed by selecting the best model out of the five obtained through cross validation. Note that training-validation was performed on a mixture of real and synthetic images, and testing was performed on a completely different test set of mixed images. Only one (randomly selected) real image in the test set was used to generate synthetic images, but the generation process ensured that latter were significantly different from the real ones.

### 2.6. Experiments and Evaluation

**Figure 4:**
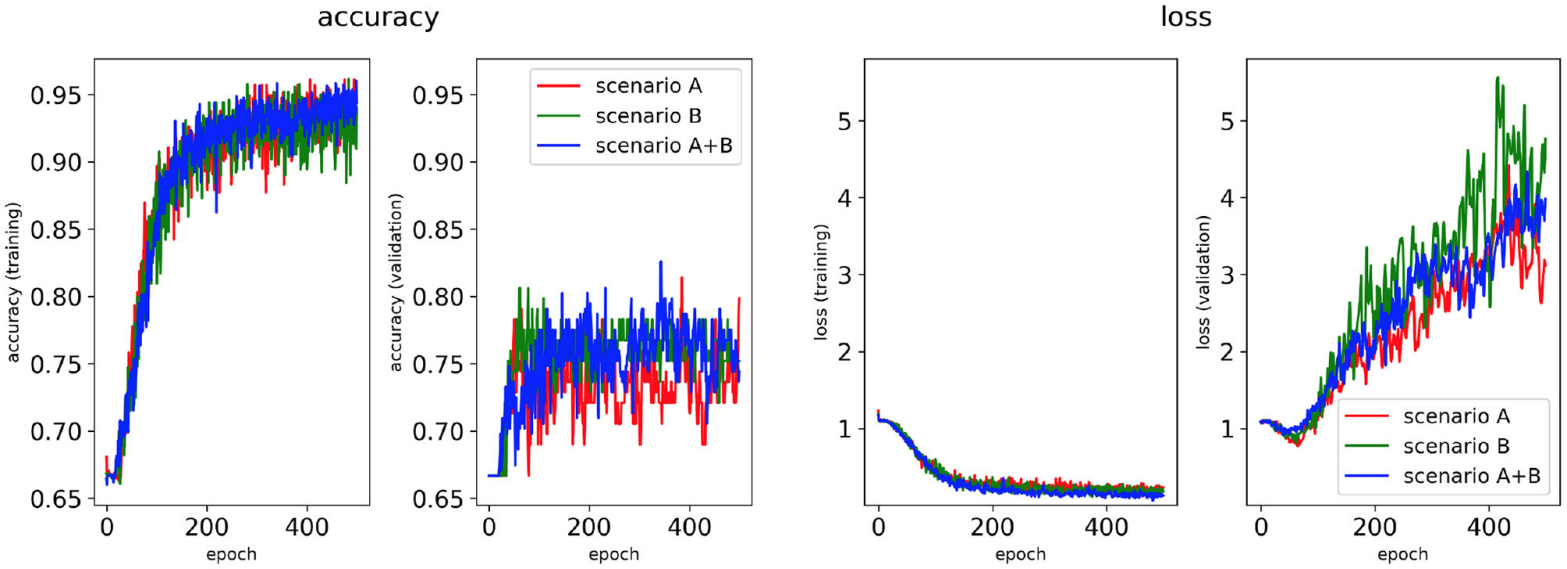
Network performance for the minimum percentage labelling method without class weighting technique. Plots of accuracy and loss (left training, and right validation) are shown for the best classifier trained on (1) Scenario A, (2) Scenario B, (3) Scenario A+B. Horizontal axis shows the epoch size from 0 to 500.

**Figure 5:**
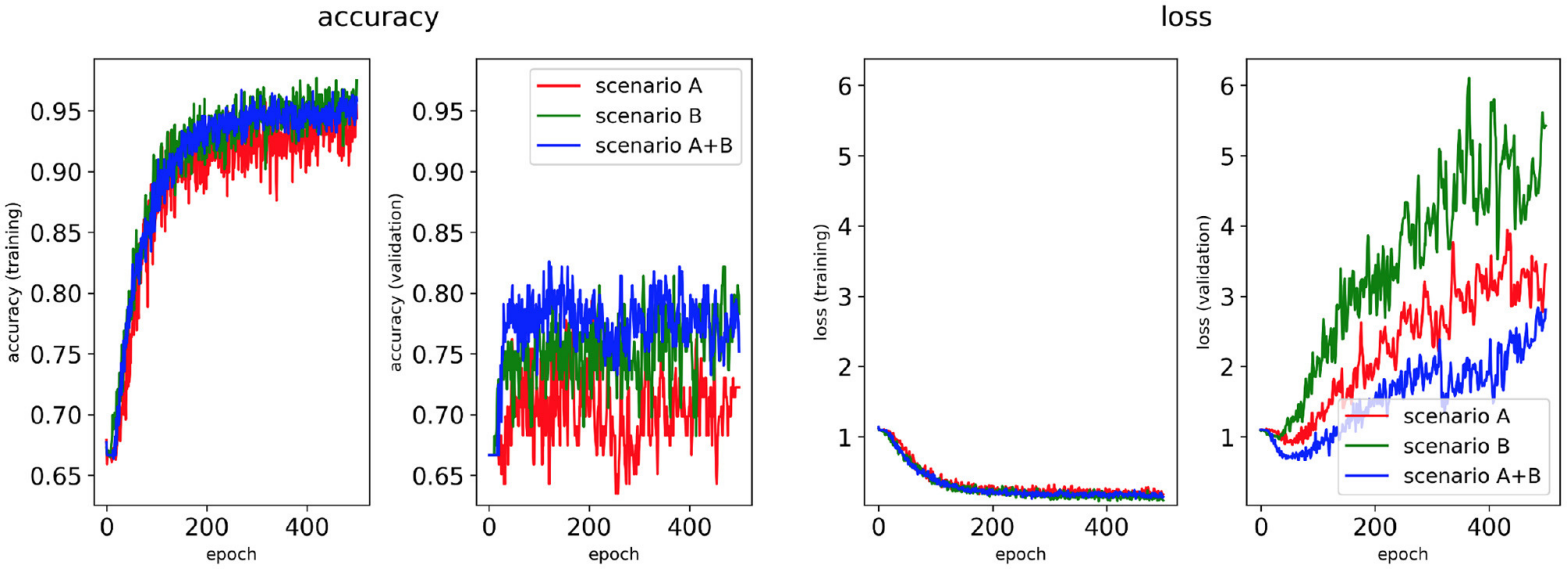
Network performance for the class availability labelling method without class weighting technique. Plots of accuracy and loss (left training, right validation) are shown for the best classifier trained on (1) Scenario A, (2) Scenario B, (3) Scenario A+B. Horizontal axis shows the epoch size from 0 to 500.

**Figure 6:**
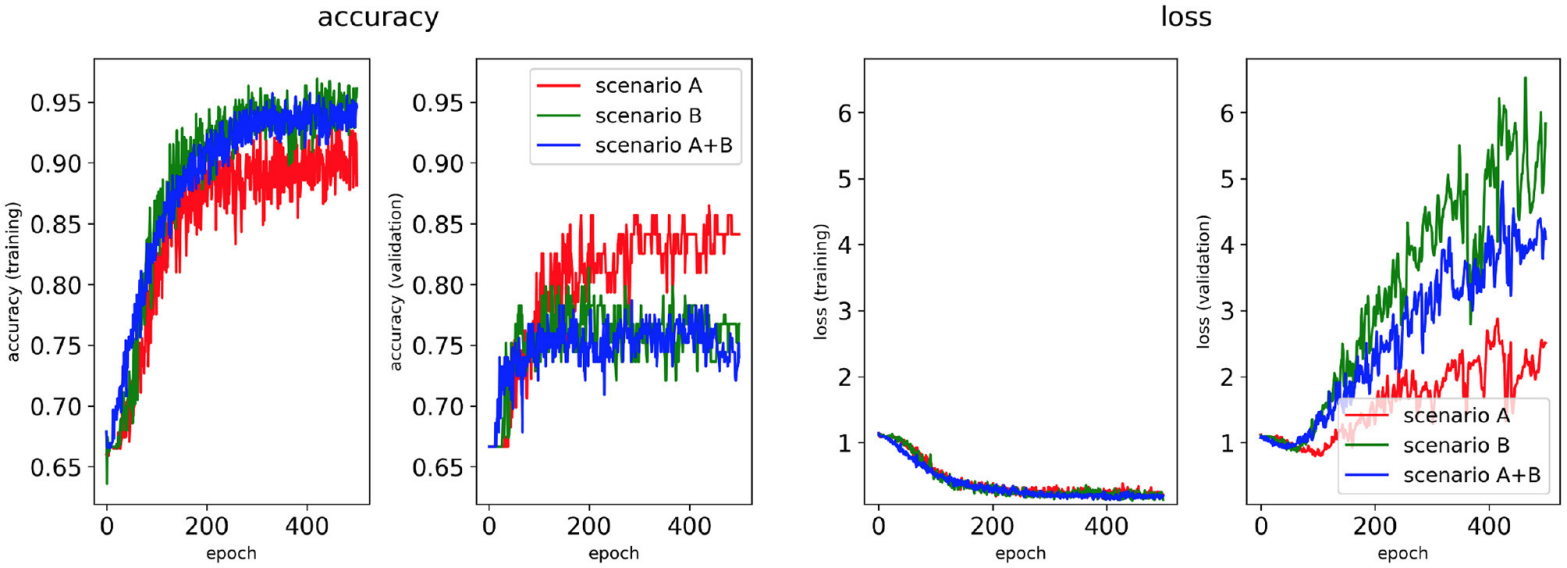
Network performance for the minimum percentage labelling method with class weighting technique. Plots of accuracy and loss (left training, right validation) are shown for the best classifier trained on (1) Scenario A, (2) Scenario B, (3) Scenario A+B. Horizontal axis shows the epoch size from 0 to 500.

**Figure 7:**
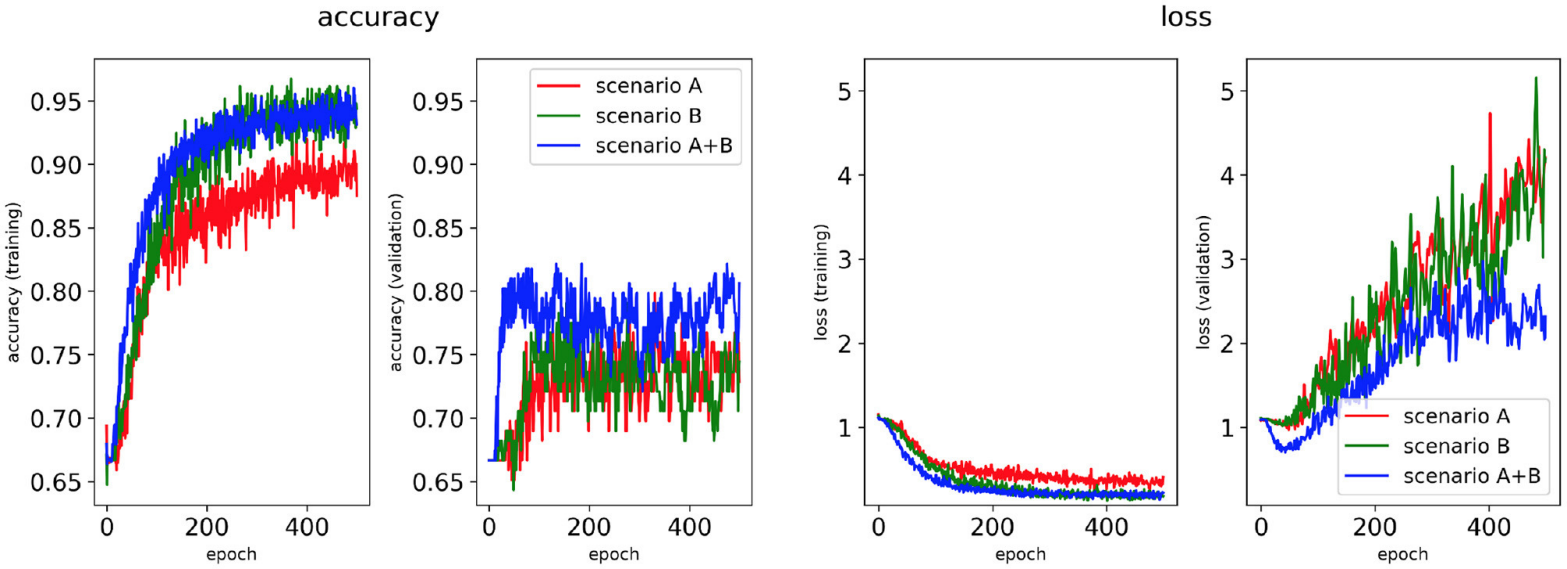
Network performance for the class availability labelling method with class weighting technique. Plots of accuracy and loss (left training, right validation) are shown for the best classifier trained on (1) Scenario A, (2) Scenario B, (3) Scenario A+B. Horizontal axis shows the epoch size from 0 to 500.

**Figure 8:**
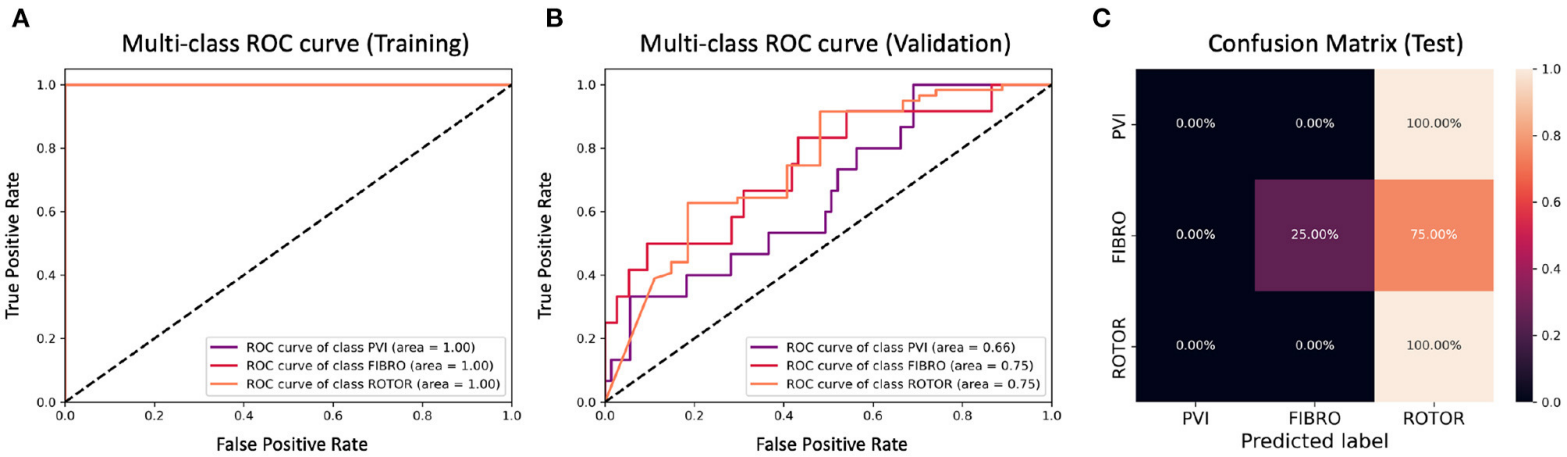
ROC curves and confusion matrix for classifier trained on Scenario A employing the minimum percentage labelling method without the class weighting technique (cf. Figure 4–Table 2 first row). The ROC curves show the performance of single-class classifications in training **(A)** and validation **(B)** based on true positive and false positive rates. The confusion matrix **(C)** shows the performance on the test set of the same classifiers. Values on the diagonals correspond to the percentage of labels predicted successfully, while others represent the percentage of unsuccessful predictions.

**Figure 9:**
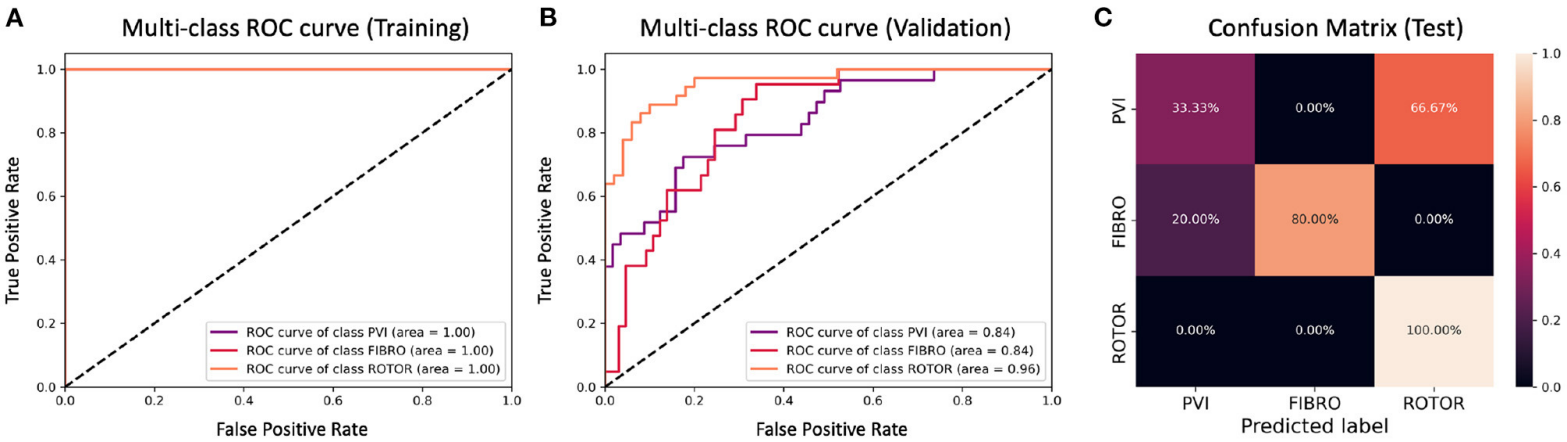
ROC curves and confusion matrix for classifier trained on Scenario A employing the class availability labelling method without the class weighting technique (cf. Figure 5–Table 2 third row). The ROC curves show the performance of single-class classifications in training **(A)** and validation **(B)** based on true positive and false positive rates. The confusion matrix **(C)** shows the performance on the test set of the same classifiers. Values on the diagonals correspond to the percentage of labels predicted successfully, while others represent the percentage of unsuccessful predictions.

**Figure 10:**
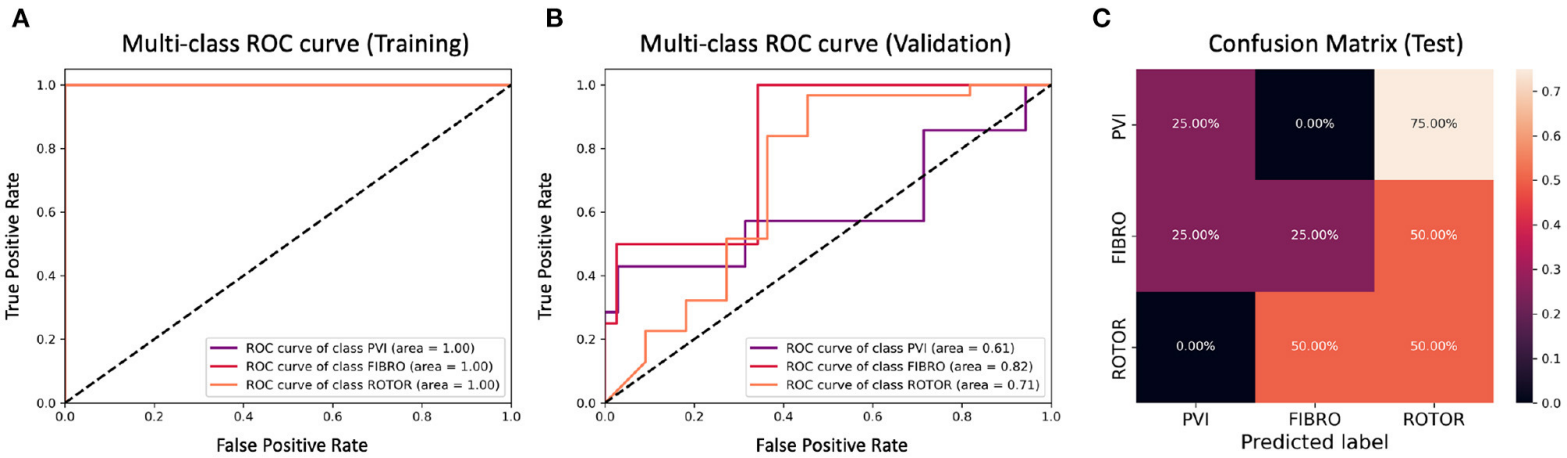
ROC curves and confusion matrix for classifier trained on Scenario A employing both the minimum percentage labelling method and the class weighting technique (cf. Figure 6–Table 2 second row). The ROC curves show the performance of single-class classifications in training **(A)** and validation **(B)** based on true positive and false positive rates. The confusion matrix **(C)** shows the performance on the test set of the same classifiers. Values on the diagonals correspond to the percentage of labels predicted successfully, while others represent the percentage of unsuccessful predictions.

**Figure 11:**
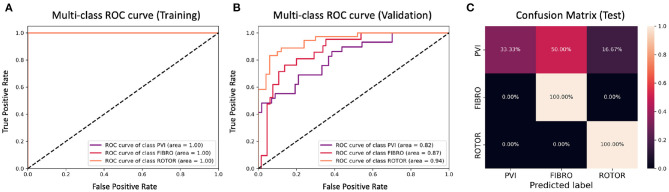
ROC curves and confusion matrix for classifier trained on Scenario A employing both the class availability labelling method and the class weighting technique (cf. Figure 7–Table 2 fourth row). The ROC curves show the performance of single-class classifications in training **(A)** and validation **(B)** based on true positive and false positive rates. The confusion matrix **(C)** shows the performance on the test set of the same classifiers. Values on the diagonals correspond to the percentage of labels predicted successfully, while others represent the percentage of unsuccessful predictions.

Four classifiers for each AF scenario were trained using: (i) the minimum percentage labelling method and without class weighting, (ii) the class availability labelling method and without class weighting, (iii) the minimum percentage labelling method and with class weighting, (iv) the class availability labelling method and with class weighting. These were evaluated using accuracy and loss plots (Figures 4–**7**), as well as precision and recall metrics (Supplementary Figures 4–7), ROC curves for training and validation sets and confusion matrices for test sets (**Figures 8**–**11**).

## 3. Results

### 3.1. Network Performance on Training and Validation Set

Performance of the CNN trained on 2D LA tissue models with AF simulated according to the three scenarios (A, B, and A+B) was evaluated for labels assigned using the minimum percentage method (Figures 4, **6**) and the class availability method (Figures 5, **7**). The classifiers were trained first without the class weighting technique (Figures 4, 5), and then employing the latter to provide a more balanced dataset in the training (Figures 6, 7). All figures show the learning curve representing CNN classification accuracy and loss in each study. Additional plots for precision and recall for each of these cases are provided in Supplementary Figures 4–7. For each combination of scenarios and labelling methods, we trained five different models using fivefold cross-validation (see Methods) and presented results for the best-performing of these models in terms of the highest validation accuracy achieved over training. Table 2 provides a summary of all classifier performances, showing that the accuracy reaches 96–97% during training and 79–86% during validation. Figures 8–**11** also illustrate the performances in training and validation for Scenario A using ROC curves.

**Table 2:** Training and validation accuracy of the best classifier for each scenario.

**Scenario**	**Labelling method**	**Class weighting**	**Model accuracy**	
			**Training**	**Validation**
Scenario A		X	96.88%	81.39%
	min percentage	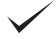	96.49%	86.50%
			
		X	96.88%	79.36%
	class availability	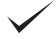	97.67%	79.84%
		X	97.49%	80.62%
	min percentage	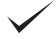	96.92%	81.40%
Scenario B		X	97.69%	82.17%
	class availability	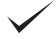	97.11%	79.84%
		X	96.61%	82.56%
	min percentage	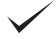	96.22%	78.68%
Scenario A+B		X	96.71%	82.56%
	class availability	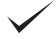	96.71%	82.17%

*Employment of different labelling methods and use of the class weighting technique are reported*.

#### 3.1.1. Minimum Percentage Labelling Method

Figures 4, 6 show accuracy and loss during training and validation for 2D LA tissues labelled using the minimum percentage method with and without applying class weighting to the loss function. In both cases, the training accuracy reaches over 95% for all scenarios and the validation accuracy is generally over 80% (Table 2). A rapid rise in accuracy and drop in loss after very few epochs can be seen for all scenarios, but the loss curve rises up again after ~50 epochs, a sign that the network might start to overfit. However, curves for accuracy in training and validation both reach a plateau at this stage. The CNN classifier performance in all scenarios is generally similar, although the network trained on Scenario A with class weighting (Figure 6) somewhat outperforms other classifiers, reaching accuracy of 86.50% during validation. The ROC curves for this scenario (Figures 8B, **10B**) are similar for the classifiers trained with (AUC = 71.3%) and without (AUC = 72%) class weighting, but there is a larger variation between single class performances in the latter case (**Figure 10B**).

#### 3.1.2. Class Availability Labelling Method

Figures 5, 7 show accuracy and loss during training and validation for 2D LA tissues labelled using the class availability method with and without applying class weighting to the loss function. In both cases, the training accuracy reaches over 95% for all scenarios, and the validation accuracy is also about 80% across the three scenarios. The same overfitting behaviour is observable after a few epochs in the loss function. The ROC curves illustrated for Scenario A (Figures 9B, 11B) are similar between the classifiers trained with (AUC = 87.67%) and without (AUC = 88%) class weighting, but the variation across classes is significantly lower and single class performance is significantly higher when compared to the respective values for the minimum percentage labelling models (Figures 8B, 10B).

### 3.2. Network Performance on Unseen Test Set

The CNN classifiers trained on 2D LA tissue models with simulated Scenario A, labelled using the minimum percentage and class availability techniques, were tested on a small unlabelled dataset of 2D LA images. The confusion matrix for the classifier trained using the minimum percentage labelling technique and no class weighting (Figure 8C) shows that generally the CNN learns to correctly predict the rotor-based CA strategy, and mislabels the other two classes as Rotor (in all cases for PVI). This is likely due to the predominance of rotor labels (see Table 1, min percentage row), which makes the classifier heavily biased toward this class. This imbalance in the training dataset was the primary reason for the introduction of the second labelling method. The behaviour is similar when using the class weighting technique (Figure 10C), but in this case there is slightly less variation between classes.

A significant improvement in testing was achieved by using the CNN classifiers trained on 2D LA tissues labelled using the class availability technique (Figures 9C, 11C). Here the values of prediction probability across the diagonal are generally higher than the external ones, which indicates a larger number of correct predictions. Although the Rotor class is still predominant (the rotor-based CA strategy is always correctly predicted in both classifiers with and without class weighting), the probability of correct predictions for the PVI and fibrosis-based CA strategies is relatively high. The predictions for the classifier with class weighting are superior (Figure 11C), with 100% success in predicting Rotor and Fibro classes, and the misclassification mostly found in distinguishing between PVI and Fibro classes. This case is further illustrated in Table 3 which provides an insight into the classification output of this CNN. A similar table including predictions obtained by the classifier trained using the minimum percentage labelling method and class weighting (cf. Figure 10C) Is shown in Supplementary Table 1.

**Table 3:** Table of test set predictions given by classifier trained on Scenario A using class availability labelling technique and class weighting (cf. Figure 7–Table 2 fourth row–Figure 11).

**Id**	**Total labels**	**Unique label**	**Predictions**		
			**PVI**	**FIBRO**	**ROTOR**
*P*10_*scenarioA*	PVI	PVI	0.3201	0.2381	0.4418
*P*02_*scenarioA*	FIBRO,ROTOR	FIBRO	0.3596	0.5352	0.1052
*OIR*_*scenarioA*	PVI,FIBRO,ROTOR	PVI	0.2839	0.4492	0.2668
38*C*_*scenarioA*	FIBRO	FIBRO	0.1917	0.4070	0.4014
*s*_26*F*_*scenarioA*	PVI	PVI	0.3997	0.4124	0.1879
*s*_11*F*_*scenarioA*	ROTOR	ROTOR	0.2628	0.0759	0.6613
*s*_9*F*_*scenarioA*	PVI,FIBRO,ROTOR	PVI	0.3552	0.4212	0.2236
*s*_3*I*_*scenarioA*	PVI,FIBRO,ROTOR	PVI	0.4284	0.4216	0.1500
*s*_10*F*_*scenarioA*	FIBRO,ROTOR	FIBRO	0.3968	0.4015	0.2017
*s*_9*G*_*scenarioA*	FIBRO,ROTOR	FIBRO	0.3691	0.4409	0.1899
*s*_4*I*_*scenarioA*	FIBRO,ROTOR	FIBRO	0.4020	0.4162	0.1818
*s*_19*F*_*scenarioA*	PVI	PVI	0.4334	0.2922	0.2745

*The Total Labels column contains all the successful strategies identified through the simulations, the Unique Label column shows the final ground truth label assigned based on class availability technique*.

*The Predictions column contains the classification output for each class*.

The confusion matrices show that the classifier may make better predictions between two classes, specifically Rotor vs. other classes. Hence, we also performed binary classifications, using CNNs with class weighting and both labelling methods, trained for the following combinations of labels (successful CA strategies): Rotor-Fibro, Rotor-PVI, PVI-Fibro (see Supplementary Figures 2, 3).

## 4. Discussion

This work builds upon previous proof-of-concept results that deep neural networks can learn from computational simulations of atrial electrical activation, to identify CA strategies (Muffoletto et al., 2019). This earlier study was based on atrial simulations run on synthetic 2D tissues with simple, randomly assigned geometric structures representing fibrosis. The current study advances this approach to utilise patient-specific imaging data and make personalised predictions of CA strategies. Specifically, we build a CNN and train several classifiers that, after careful clinical validation, can help tailor CA strategies to individual AF patients, which is currently one of the ultimate aims of research in the field of AF. To achieve this, we first process a large LGE-MRI dataset (Section 2.0.1) that we also augment with a synthetic dataset (section 2.0.2) to provide more variability in the CNN training. We then train the CNN classifiers using a combination of real and synthetic images and labels produced by image-based models of AF and CA therapy. We consider three main CA strategies (PVI, rotor-based and fibrosis-based) and also two AF scenarios that represent early- and late-stage AF. The advancements on our previous work (Muffoletto et al., 2019) include: (i) the use of real patient images to generate atrial models and train the CNN, (ii) the use of a wider range of simulated AF scenarios and ablation strategies to produce more accurate labels for the images, (iii) optimisation of the CNN parameters to produce higher accuracy in training and validation, and (iv) more in-depth analysis of the CNN classification between the CA strategies.

The ground truth labels for the images were assigned based on successful termination of AF by ablation in the image-based 2D LA models. If multiple successful CA strategies for the same LA model were identified, we used the minimum percentage (in terms of ablated tissue) method which prioritises less invasive CA strategies. Classification results (Figure 4) show that this method achieves similar performance across two different AF scenarios (A and B) and their combination (A+B). Values for the accuracy during validation on Scenario A (81.39%) and Scenario A+B (82.56%) are particularly promising but when analysing the ROC curve in Figure 8B, we can observe a high variation of the AUC values between the classes. This is further reflected in the confusion matrix for this classifier (Figure 8C), which predicted rotor-based CA as the most successful strategy even in cases where simulations showed that PVI and fibrosis-based CA were more suitable strategies.

For this reason, the same classification experiments were repeated using a different labelling technique, referred to as class availability method, which prioritises the assignment of ground truth labels based on ranking of least common successful strategies in the simulations (in the order: PVI, Fibro, and Rotor). This technique allowed us to produce a more balanced training dataset and to achieve a training accuracy of 96–97% and a validation accuracy of 79–82% across scenarios (Figure 5 and Table 2). The ROC curve (Figure 9B) shows higher AUC values for all classes compared to the first labelling method, and a better performance across them, which is also visible in the confusion matrix for the testing (Figure 9C), where values of prediction per class are promising, except for PVI that is often mislabelled as Rotor. Note that PVI had low success rate in the 2D atrial model simulations, which may be due to the PVs contributing to AF mechanisms primarily via ectopic triggers, rather than via rotors considered in the current study. Expanding the models to account for the PV triggers, and hence naturally increasing the number of images labelled as PVI class, should improve the prediction for this class.

To provide a more balanced distribution of labels (successful CA strategies) in the dataset, we used two different approaches: (1) a stratified 5-fold cross validation which can be considered as a valid alternative to bootstrapping (Kohavi, 1995), and (2) weighting of the loss function based on the percentage of samples in each class, a common approach for unbalanced datasets (Thai-Nghe et al., 2010; Rosenberg, 2012; Huang et al., 2013). The addition of class weighting to the minimum percentage labelling method doesn't lead to a clear advantage (Figure 9C). However, the addition of class weighting to the class availability labelling method shows a substantial improvement in the prediction of Rotor and Fibro classes (100%) (Figure 11C).

Although we have implemented several techniques to improve balance of the dataset, one of the limitations of this study remains the high variation of predictive ability of the classifiers across the three classes. In particular, the presence of such a low success rate of the PVI class in the real set may be explained by the facts that (i) the PVs in LA-SUM disks (Williams et al., 2017) are small and cannot sustain rotors around them, and (ii) PVs contribute to AF mechanisms mainly by sustaining its triggers that were not accounted for in our models. Further work should include the collection of more patient-specific data and its employment for generation of more synthetic data that specifically target the enhancement of the PVI and fibrosis subsets.

It would also be of crucial importance to apply more detailed 3D atrial models that include MRI-derived atrial geometries and region-specific electrophysiology (Morgan et al., 2016; Varela et al., 2017; Roy et al., 2018, 2020). Although the rotor dynamics shows qualitative similarities between 2D and 3D atrial models, such as anchoring of the rotors to large fibrotic patches (Supplementary Figure 8), detailed 3D models will be needed to achieve true patient-specific predictions. The reason for using 2D atrial simulations in our study was the efficiency in providing the needed proof of concept: (i) running 3D atrial simulations for several hundred cases would take years of simulations on a supercomputer, which would be a gross misuse of computational power; (ii) standardised 2D unfolded atrial images allowed us to easily generate a large number of additional synthetic images, which is crucial for training CNNs. Moreover, (iii) our previous work has shown that 3D atrial wall thickness is distributed relatively evenly in the LA outside of PVs and slow conduction in fibrotic areas is the main determinant of the rotor dynamics (Roy et al., 2018). Hence, image-based 2D atrial models provided a sensible balance between realistic atrial details (such as fibrotic distribution) and computational efficiency (primarily the ability to run a large number of simulations and train the CNN).

Another limitation includes the size of the unseen test set, which was kept small to avoid the subtraction of data from the training set. More substantial and more balanced training and test sets should enable achieving higher accuracy in the classification using the minimum percentage labelling method, which is more clinically relevant, as it encourages less invasive CA strategies. The second labelling technique based on the class availability was introduced to even out the distribution of the three classes, and should be considered as a proof of concept. Finally, it's worth noting that a significant proportion of CA simulations were unsuccessful (101), and other CA strategies may need to be considered in the future for such cases.

Boyle et al. (2019) have identified patient-specific targets for ablation from LGE-MRI based computational models of the 3D atria and demonstrated the feasibility of this approach to guide patient treatment in a prospective study of 10 patients. The locations of rotors sustaining AF in their models was strongly correlated with the location of fibrotic areas identified from LGE-MRI. Our group have also reported similar results (Roy et al., 2020). Such studies provide further evidence to the value of research on rotor-based and fibrosis-based to AF ablation, as well as of computational models in such research. The main novelty of the current study is the application of CNNs to make predictions from a combination of imaging and modeling data. While image-based modeling can provide useful information about structure-function relationships during AF, its downsides include (i) huge computational power needed to simulate multiple AF scenarios in the detailed 3D atrial models, and (ii) the need to rerun the models each time novel data is integrated into them, which makes the application of models in a clinical setting impractical. The CNN can overcome these limitations, and - after careful validation and integration of clinical data—could provide a fast and flexible tool to help predict ablation strategies for a large patient population.

PVI is the cornerstone of AF ablation procedures. However, while PVI has proven highly effective in treating paroxysmal AF patients, ablation of more challenging persistent AF patients often requires additional lesions beyond PVI; even after successful termination, long-term success of PVI in persistent cases is highly suboptimal. Hence, the search for novel ablation targets has been a focus of research for two decades, with both rotor and fibrosis based ablations showing the greatest promise (Parameswaran et al., 2021), and considering them as potential candidates in our study along with PVI is a logical and timely step in this research. In regards to fibrosis-based ablation, it is worth noting that Yang et al. (2017) have shown that there was little long-term benefit in substrate modification compared to PVI in non-paroxysmal AF patients. However, the study provides no direct evidence that their approach targeted fibrosis, as they targeted low-voltage areas that are known to have poor spatial correlation with fibrotic areas. In regard to rotor-based ablation, Tilz et al. (2020) have shown that PVI had similar effectiveness to FIRM-guided ablation in paroxysmal AF patients. However, (i) their study provided no evidence that the same applies to persistent AF patients and (ii) FIRM is known for its limitations in identifying rotors, and therefore the study points to the limitations of the FIRM approach specifically, rather than of the rotor-based approach in general. Hence, the question on the role of ablating fibrosis and rotors in persistent AF patients remains open; results of the ongoing DECAAF II trial should provide more direct evidence for the former.

The strength of the modelling approach used in this study is in its ability to test ablation procedures that target rotors and fibrosis with tractable properties – rather than markers believed to have some correlation with the presence of rotors (FIRM) and fibrosis (low-voltage areas). While differences between modelling and clinical data are inevitable, modelling enables exploring promising approaches to AF therapy in depth that may not be achievable in a purely clinical setting. Specifically in our study, this allows us to (i) simulate ablation of rotors and fibrosis with tractable properties and (ii) provide a proof-of-concept that a CNN can be trained based on a combination of structural (patient MRI) and functional (modelling) data to make predictions about suitable ablation strategies. The model in this case only provides a label (a suitable CA strategy) for a patient image, which is used to train the CNN. This builds confidence in the computational approach – which can then be used for images labelled using data from patients (should such data be available), and help make clinically valid predictions. Moreover, the approach is restricted to the three strategies considered in the current study, and can include any other promising ablation strategies, with the CNN helping to choose the most suitable one for each patient. It is important to stress that all stages of this approach need to undergo careful clinical validation before it can be applied in a clinical setting. Importantly, any additions to the gold-standard PVI procedure should be treated with extra care and be aimed at improving, rather than complicating the existing clinical approaches.

**Figure 12:**
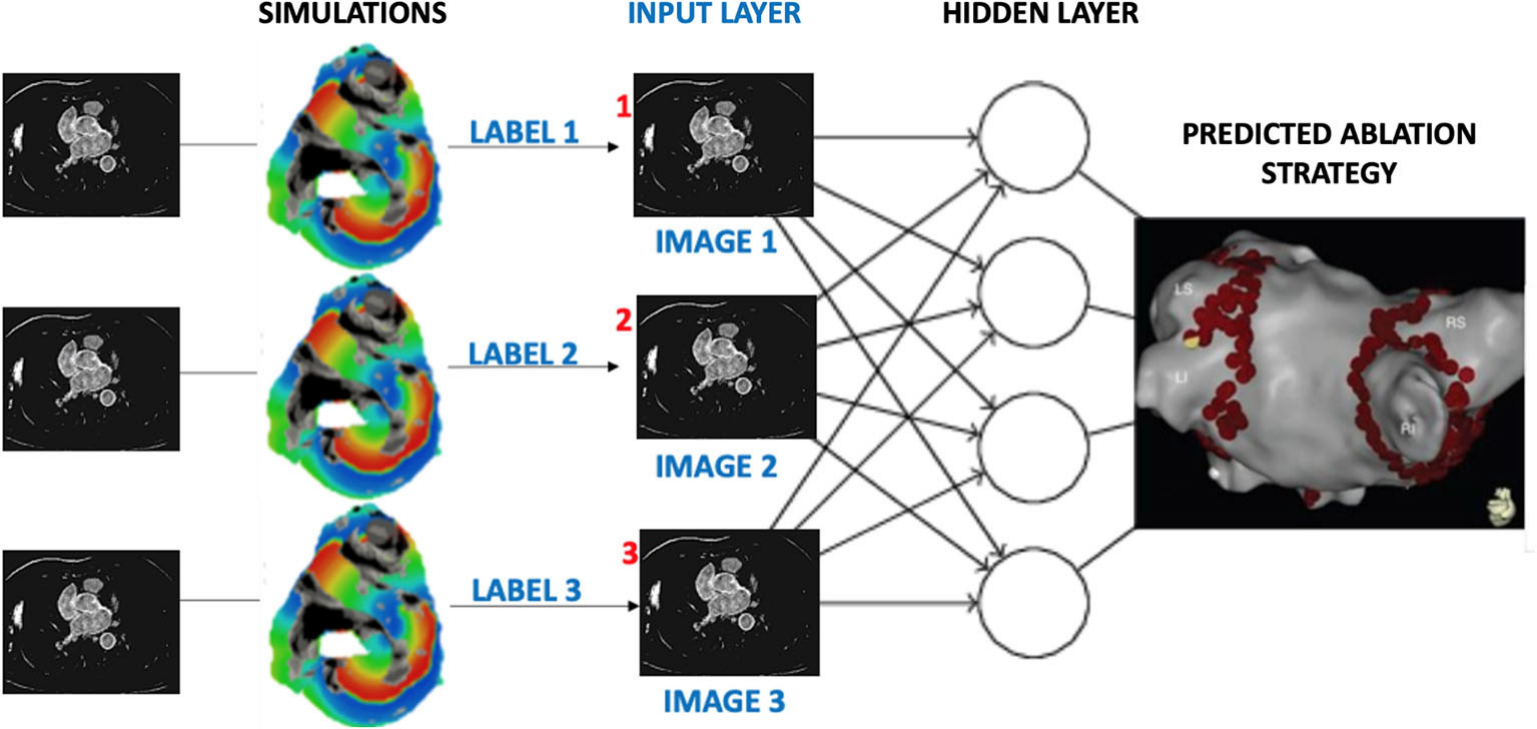
Proposed workflow for assigning labels to patient MR images based on image-derived 3D simulations of atrial electrical activation. Similar to the current study, labels would be given to each patient dataset according to the rate of success for several AF ablation scenarios. Once passed through the layers of a CNN, the output would be the prediction of a suitable CA strategy for a given patient.

In conclusion, this work proposes a unique novel approach to personalisation of AF ablation therapy, which is based on a combination of patient imaging, image-based modelling and deep learning. The importance of a patient-specific approach to AF therapy is being increasingly recognised (Cochet et al., 2018; Roney et al., 2018; Parameswaran et al., 2021), and we believe that such advanced image-based computational technologies will play an important role in achieving such personalisation in the future. Out results show that deep neural networks can provide a sensible approach to predict a suitable ablation strategy for the considerably high number of people suffering from AF. Ultimately, our aim would be to directly apply a similar deep learning approach to 3D datasets that combine information from volumetric patient MRI scans with the image-derived 3D atrial model simulations, as illustrated in Figure 12. Additional validation for the network predictions should be performed against outcomes of actual CA procedures applied to the patients, building up further toward clinical application of this approach.

## Data Availability Statement

The original contributions presented in the study are included in the article/Supplementary Material, further inquiries can be directed to the corresponding author/s.

## Author Contributions

MM and OA conceived and designed the study, and drafted the manuscript. MM, AQ, and AZ substantially contributed to data analysis and computations. OA and PB substantially contributed to the interpretation of the results. LM, XF, JZ, and AR contributed to data analysis and computations. All authors have also approved the final version to be published while agreeing to be accountable for all aspects of the work in ensuring that questions related to the accuracy or integrity of any part of the work are appropriately investigated and resolved. All authors have made significant contributions to this study.

## Conflict of Interest

The authors declare that the research was conducted in the absence of any commercial or financial relationships that could be construed as a potential conflict of interest.
