# Supplementary material for: Toward Patient-Specific Prediction of Ablation Strategies for Atrial Fibrillation Using Deep Learning
Source: Front Physiol. 2021 May 26;12:674106. doi: 10.3389/fphys.2021.674106 (PMC8187921; doi:10.3389/fphys.2021.674106)
Supplement: Supplementary file 1 [file Data_Sheet_1.PDF]

Towards Patient-Specific Prediction of Ablation Strategies for Atrial Fibrillation using Deep Learning

Supplementary Material

1 SUPPLEMENTARY DATA

Two videos of 2D LA tissue model simulations illustrating successful and unsuccessful CA. These represent different LA-SUM disks ablated with fibrosis-based CA strategy, thus points around fibrotic patches are ablated in series every 10ms. First video (Video S1) shows termination of AF after 1350ms, while in another one (Video S2) CA is not successful and the simulation ends at the threshold time of 2000ms. The same colour scheme as seen in Fig. 2-3 of the main manuscript is utilised in the videos.

2 SUPPLEMENTARY TABLES AND FIGURES

2.1 Figures

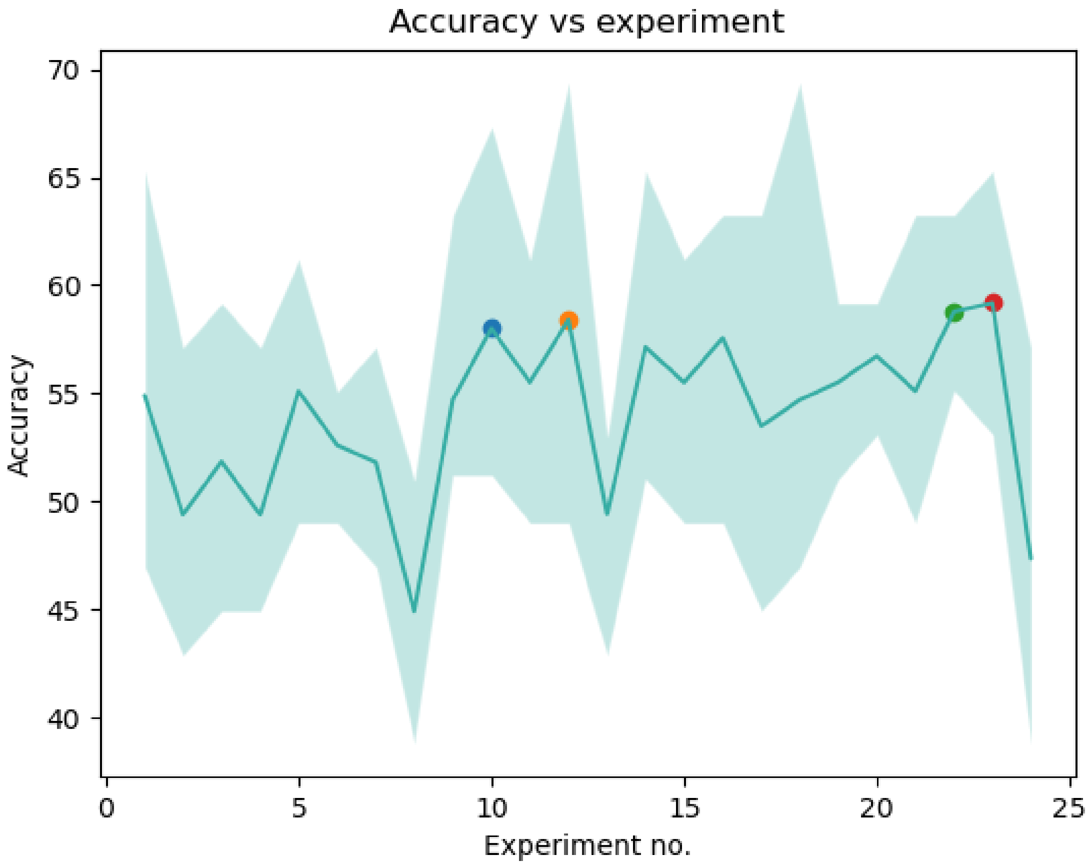

Figure S1: Changes in performance across experiments depending on CNN architecture and hyperparameters. These were carried out on the real 2D LA tissue model set only, and using a four-class classification that included the 2D LA models with no successful ablation. Hence, lower accuracy was achieved compared to the three-class classification results shown in main manuscript. The main line shows the average accuracy achieved over 5 folds, while the shaded region covers the range of values found within the different classifiers. The horizontal axis refers to all 24 experiments that were performed varying 1) number of conv2D layers; 2) filter size of conv2D layers; 3) dropout rate; 4) optimiser initial learning rate; 5) optimiser rate change; 6) rate of change patience. Based on the accuracy value (vertical axis), the coloured dots show that experiments 10, 12, 23 and 24 achieve higher accuracy. In most of these experiments 1) was either 3 or 4; 2) was a combination of (5,5) and (3,3) filters; 3) was set to 0.9; 4) was set to 0.0005; 5) was either 0.8 or 0.5; and 6) was either 100 or 200. The final parameters (as described in the Methods section 2.3) were set as suggested by experiment 23 which achieved average accuracy of 59.18%.

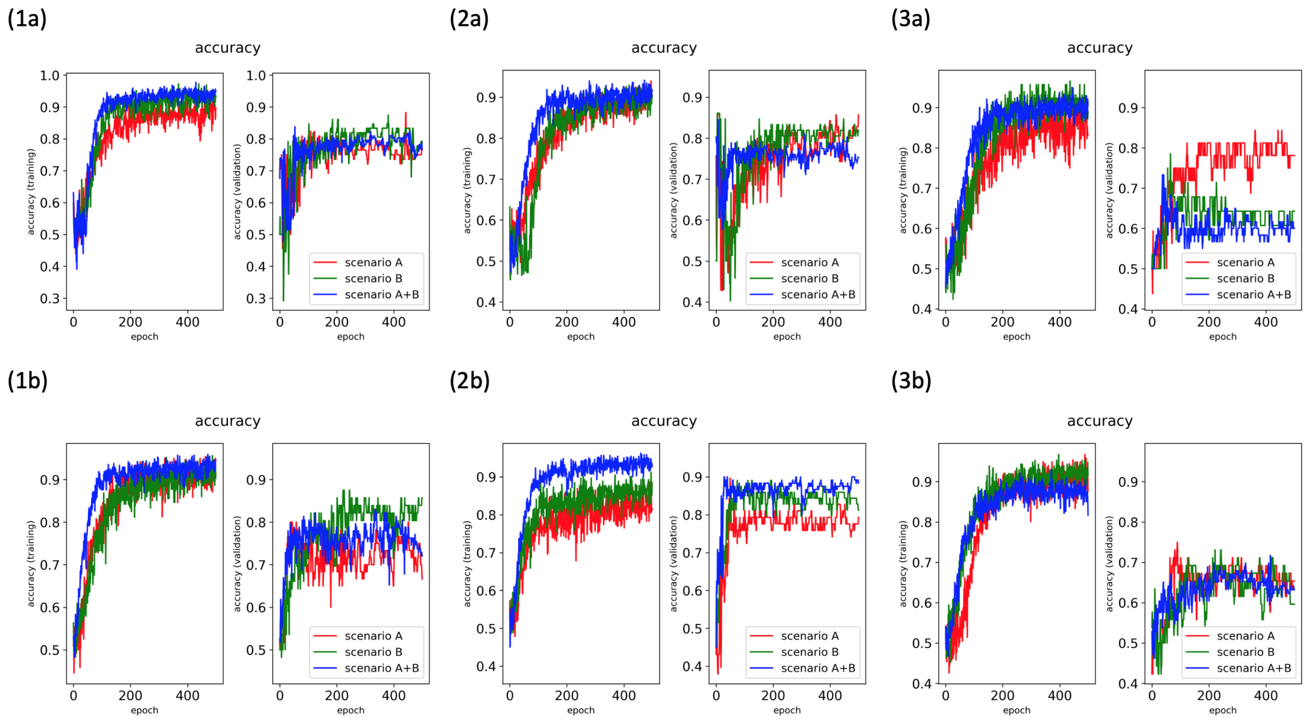

Figure S2: Accuracy in binary classifications. Similarly to results for multi-class classifications, accuracy is shown over 500 epochs for each classifier. a) and b) refer to the labelling method chosen to assign the ground truth labels while the numbers 1-3 refer to the type of classification, respectively: 1) Rotor-Fibrosis classification, 2) Rotor-PVI classification and 3) PVI-Fibrosis classification. All models employed here use the class weighting technique.

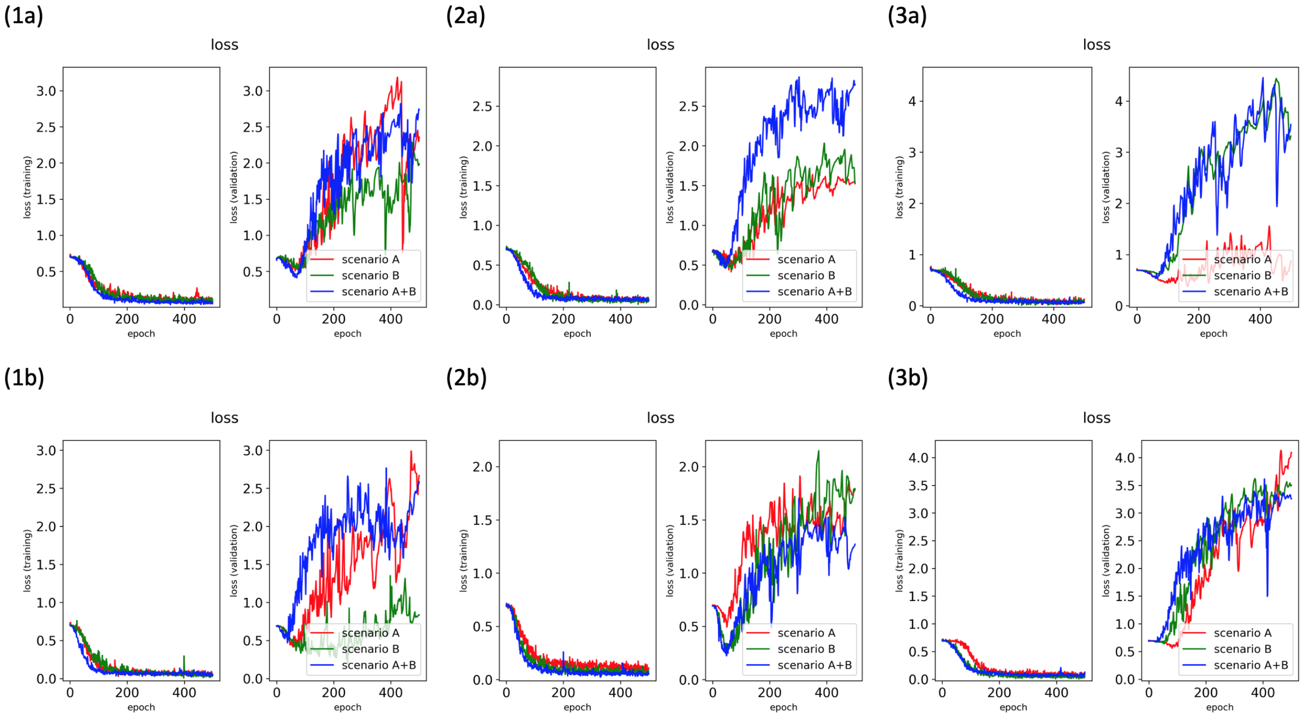

Figure S3: Loss in binary classifications. Similarly to results for multi-class classifications, loss is shown over 500 epochs for each classifier. a) and b) refer to the labelling method chosen to assign the ground truth labels while the numbers 1-3 refer to the type of classification, respectively: 1) Rotor-Fibrosis classification, 2) Rotor-PVI classification and 3) PVI-Fibrosis classification. All models employed here use the class weighting technique.

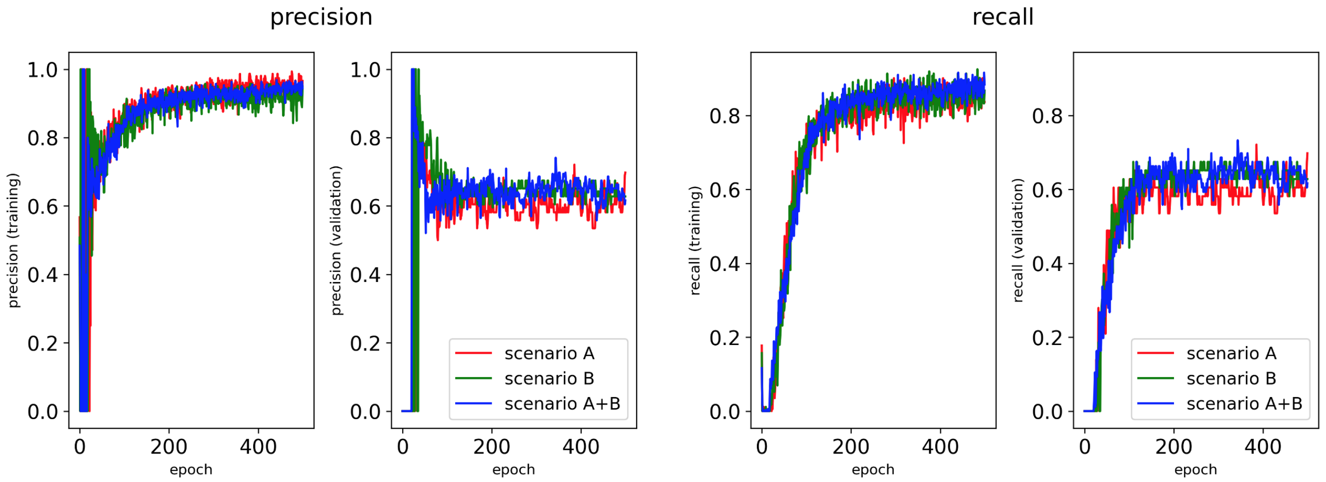

Figure S4: Network performance for the minimum percentage labelling method without class weighting technique. Plots of precision and recall (left training, right validation) are shown for the best classifier trained on 1) Scenario A, 2) Scenario B, 3) Scenario A+B. Horizontal axis shows the epoch size from 0 to 500.

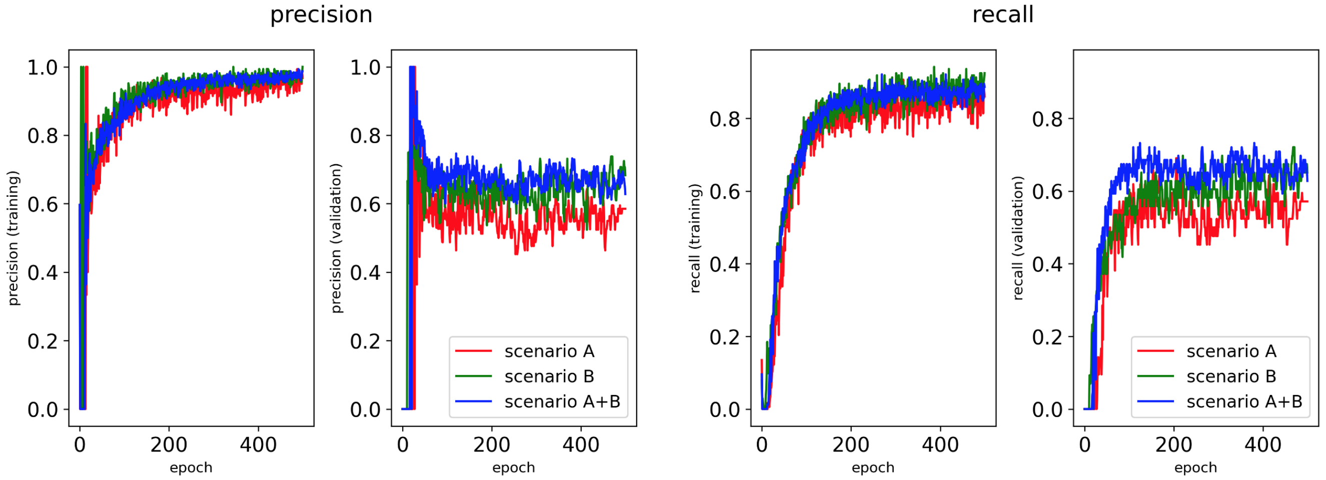

Figure S5: Network performance for the class availability labelling method without class weighting technique. Plots of precision and recall (left training, right validation) are shown for the best classifier trained on 1) Scenario A, 2) Scenario B, 3) Scenario A+B. Horizontal axis shows the epoch size from 0 to 500.

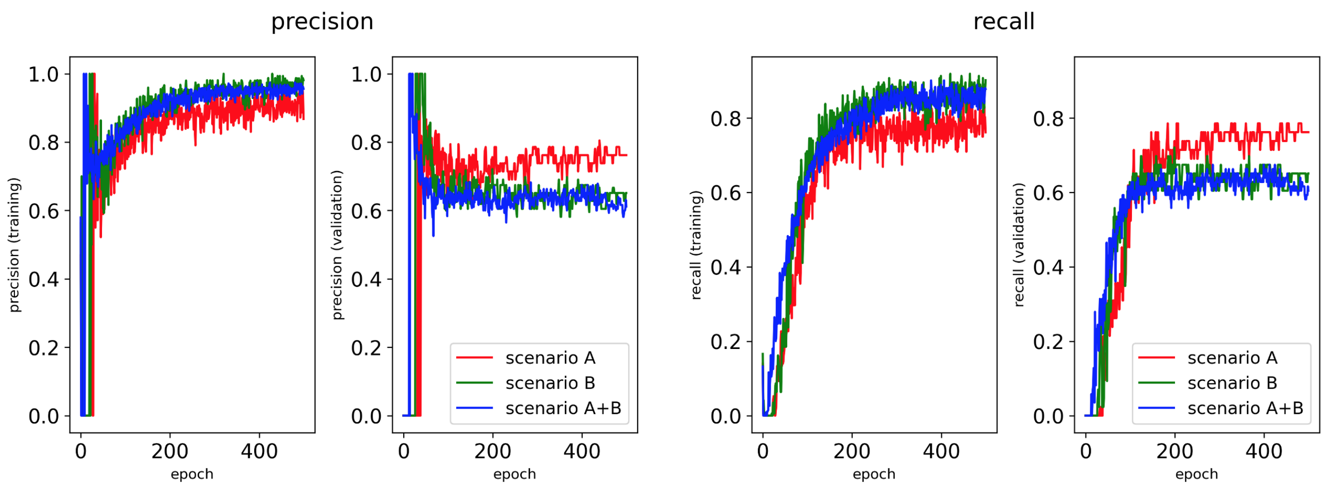

Figure S6: Network performance for the minimum percentage labelling method with class weighting technique. Plots of precision and recall (left training, right validation) are shown for the best classifier trained on 1) Scenario A, 2) Scenario B, 3) Scenario A+B. Horizontal axis shows the epoch size from 0 to 500.

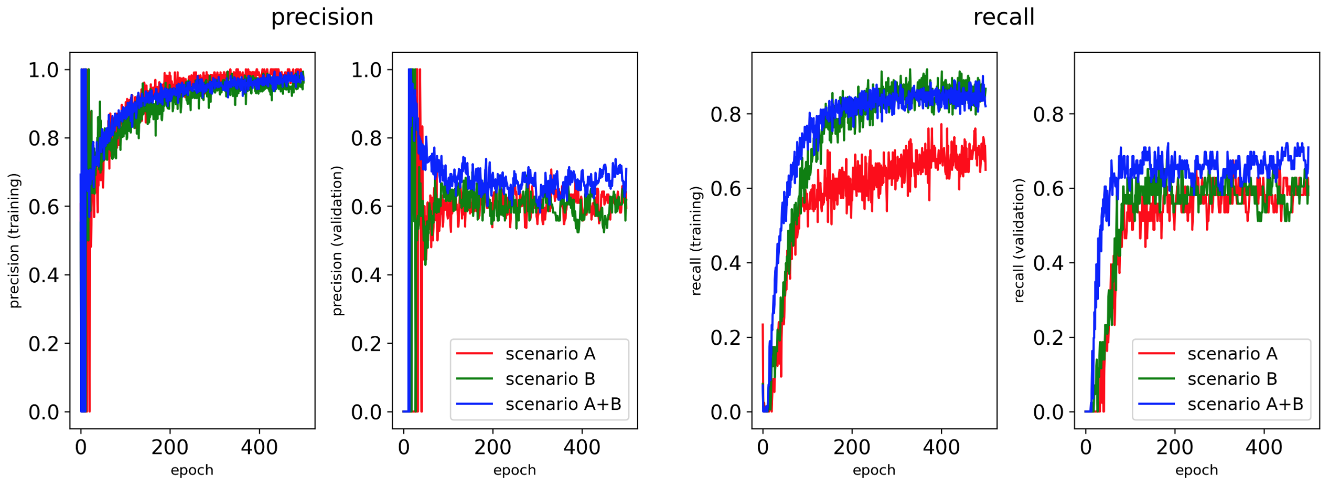

Figure S7: Network performance for the class availability labelling method with class weighting technique. Plots of precision and recall (left training, right validation) are shown for the best classifier trained on 1) Scenario A, 2) Scenario B, 3) Scenario A+B. Horizontal axis shows the epoch size from 0 to 500.

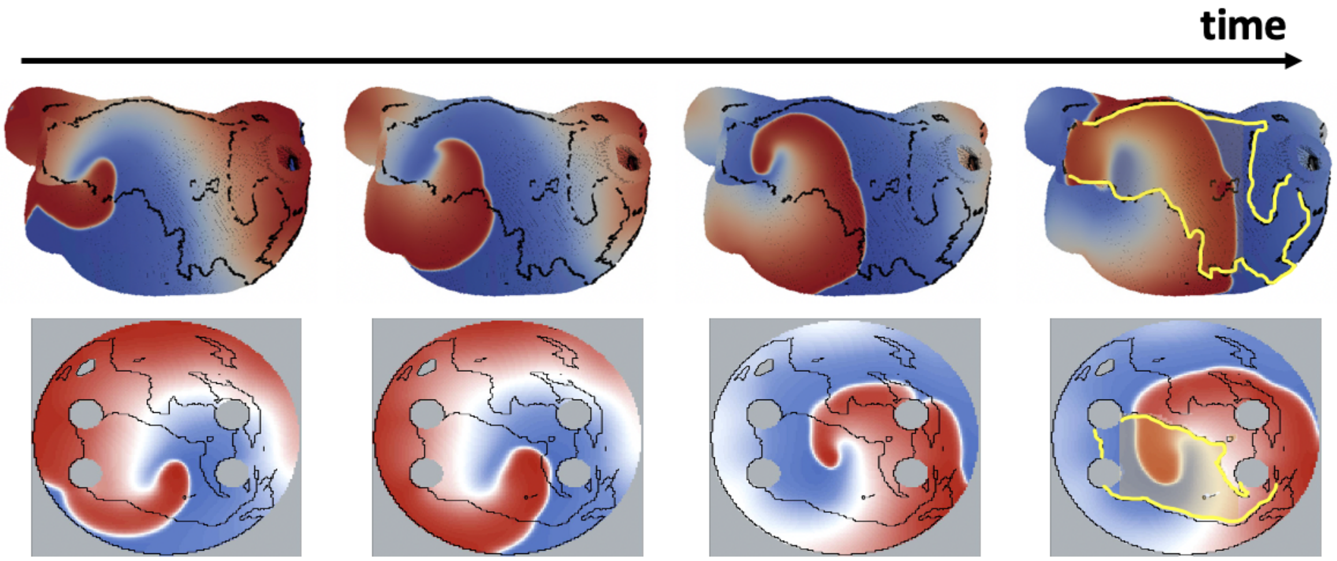

Figure S8: Comparison between the rotor dynamics simulated with image-based atrial models: (A) 3D LA and (B) unwrapped 2D LA. In both cases, the rotor anchors to the same fibrotic patch highlighted in yellow.

2.2 Tables

Table S1. Table of test set predictions given by classifier trained on Scenario A using minimum percentage labelling technique and class weighting (cf. Fig. 6 and Table 2 second row in main paper). The Total Labels column contains all the successful strategies identified through the simulations, the Unique Label column shows the final ground truth label assigned based on minimum percentage technique. The Predictions column contains the classification output for each class.

ID	TOTAL LABELS	UNIQUE LABEL	PREDICTIONS		
			PVI	FIBRO	ROTOR
$P10\_scenarioA$	PVI	PVI	0.0002	0.0000	0.9998
$P02\_scenarioA$	FIBRO,ROTOR	ROTOR	0.0000	0.9689	0.0311
$OIR\_scenarioA$	PVI,FIBRO,ROTOR	PVI	0.0501	0.0370	0.9129
$38C\_scenarioA$	FIBRO	FIBRO	0.0000	0.0002	0.9998
$s\_26F\_scenarioA$	PVI	PVI	0.9759	0.0000	0.0241
$s\_11F\_scenarioA$	ROTOR	ROTOR	0.0000	0.0000	1.0000
$s\_9F\_scenarioA$	PVI,FIBRO,ROTOR	FIBRO	0.0017	0.0000	0.9983
$s\_3I\_scenarioA$	PVI,FIBRO,ROTOR	ROTOR	0.0087	0.0011	0.9902
$s\_10F\_scenarioA$	FIBRO,ROTOR	FIBRO	0.9791	0.0000	0.0209
$s\_9G\_scenarioA$	FIBRO,ROTOR	FIBRO	0.0000	0.9990	0.0010
$s\_4I\_scenarioA$	FIBRO,ROTOR	ROTOR	0.0001	0.9907	0.0092
$s\_19F\_scenarioA$	PVI	PVI	0.0000	0.0001	0.9999
